# Maintaining vs. milking good reputation when customer feedback is inaccurate

**DOI:** 10.1371/journal.pone.0207172

**Published:** 2018-11-12

**Authors:** Behnud Mir Djawadi, René Fahr, Claus-Jochen Haake, Sonja Recker

**Affiliations:** 1 Department of Management, Paderborn University, Paderborn, Germany; 2 Institute for the Study of Labor (IZA), Bonn, Germany; 3 Department of Economics, Paderborn University, Paderborn, Germany; Shandong University of Science and Technology, CHINA

## Abstract

In Internet transactions, customers and service providers often interact once and anonymously. To prevent deceptive behavior a reputation system is particularly important to reduce information asymmetries about the quality of the offered product or service. In this study we examine the effectiveness of a reputation system to reduce information asymmetries when customers may make mistakes in judging the provided service quality. In our model, a service provider makes strategic quality choices and short-lived customers are asked to evaluate the observed quality by providing ratings to a reputation system. The customer is not able to always evaluate the service quality correctly and possibly submits an erroneous rating according to a predefined probability. Considering reputation profiles of the last three sales, within the theoretical model we derive that the service provider’s dichotomous quality decisions are independent of the reputation profile and depend only on the probabilities of receiving positive and negative ratings when providing low or high quality. Thus, a service provider optimally either maintains a good reputation or completely refrains from any reputation building process. However, when mapping our theoretical model to an experimental design we find that a significant share of subjects in the role of the service provider deviates from optimal behavior and chooses actions which are conditional on the current reputation profile. With respect to these individual quality choices we see that subjects use milking strategies which means that they exploit a good reputation. In particular, if the sales price is high, low quality is delivered until the price drops below a certain threshold, and then high quality is chosen until the price increases again.

## Introduction

Reputation systems collect and publish customer feedback to reduce information asymmetries between sellers and customers. Nowadays, online rating portals make it easy for customers to report their (dis-)satisfaction with virtually any product or service. However, customer feedback is not always accurate. On the one hand, there is ample evidence that customers fake transactions and provide dishonest feedback [1, 2]. On the other hand, assuming that this kind of strategic behavior is unintentional, customers simply may not have the required expertise to judge the service or product quality correctly and may make mistakes.

Such inaccuracies appear in a wide range of markets, ranging from hotel and restaurant services, IT (cloud) services or digital and electronic devices. For example, a customer’s negative rating for a restaurant service might be due to his inability to distinguish good from bad quality of the ingredients of the meal or to taste the exclusivity of the wine. As a result, a negative feedback may be given, even if the service provider (in this example the owner of the restaurant) delivered high quality (in terms of fresh ingredients or excellent wine). On the contrary, a restaurant with many positive ratings attracts many customers who expect to find high-quality service and who are willing to pay a higher price for the service compared to a restaurant with same service but worse reputation profile.

From a behavioral perspective, a higher sales price as a result of sufficiently many positive ratings, in turn provides incentives to cheat on customers and deliver low quality, e.g. stale meat, at a high price. If the owner of the restaurant anticipates that customers only walk in once and may make mistakes in judging the quality of the service, he might be tempted to serve low quality (e.g., old ingredients or low quality meat) and still charge a high price. Certainly, strategic behavior in quality choices is not limited to the restaurant example. One may think more generally of experience goods, i.e., goods or services whose quality cannot be observed prior to consumption. For expositional reasons we recur to the restaurant example in the subsequent discussion, although the approach is far more general. The service provider, or simply seller, is in that case the restaurant owner, while the service itself includes everything that is delivered from the service provider to the customer.

Our main research question is how inaccurate customer feedback based on unintentional mistakes influences the strategic quality choices of a single service provider. More precisely, we investigate (a) whether a seller seeks to maintain a good reputation by providing high-quality services, even if it is tempting that low quality might not be detected by the customer, or (b) whether a seller milks a good reputation and exploits the customers. Thus, we investigate the effectiveness of reputation systems to reduce information asymmetries on markets with inaccurate customer feedback.

In particular, in our study we model the rating behavior of the customer exogenously and non-strategically. Neither are customers able to react to the delivered service quality reciprocally nor are customers able to influence the pricing of the service in future periods by strategically misreporting the true quality of the delivered service.

The analysis is theoretical as well as experimental. First, we model the service provider’s strategic quality choice as a stochastic optimization problem and use a discounted Markovian decision process where states correspond to the service provider’s reputation profiles. In our model the service provider chooses to produce either high- or low-quality services and receives a sales price depending on the current reputation profile. A reputation profile consists of the last three ratings, whereby a rating is either positive or negative and the price is higher the more positive ratings appear in the reputation profile.

To motivate the dependence between sales price, hence profits, and the reputation profile one may think of a competitive market, in which all service providers have equal quality and cost structures. However, the better the reputation profile compared to the competitors, the larger the service provider’s share of the overall demand (e.g. restaurants are sorted in restaurant guides by their reputation and therefore better rated restaurants are more visible than others). So, even if one leaves the price fixed in the model, higher reputation may be translated into higher profits. To keep the analysis and the experiment tractable, we model these profit changes based on reputation profiles by keeping the demand constant for all service providers and by changing the price a single service provider may charge for the service.

To account for anonymity inherent in many markets we assume that customers are short-lived, meaning that each customer interacts only once with the service provider. This assumption avoids trustful and presumably reciprocal relationships between service providers and customers, which would definitely lead to a different strategic behavior. In the restaurant scenario, one may think of walk-in customers (probably at a touristic place). After purchase, a customer either positively or negatively evaluates the service according to predefined error probabilities. These stochastic ratings reflect the customer’s limited ability to correctly judge the provided service quality. Hence, the evaluation of the reputation system takes judgment biases and uncertainty in complex transactions into account.

Second, we design a laboratory experiment that is captured by the theoretical model. All subjects participating in our experiment play the role of the service provider and choose to produce high- or low-quality services. Four different treatments vary by the customers’ evaluation abilities to accurately rate the service quality. Besides analyzing the empirical relevance of optimal strategies derived from our theoretical model, we are able to investigate behavioral conjectures. By considering exogenous rating behavior depending on different evaluation abilities of the customer together with a sales price resulting directly from the reputation profile we are able to identify causal effects of the reputation system on the quality choices of the service provider. From a theoretical perspective a profit-maximizing service provider is expected to either always produce high-quality services or to always produce low-quality services, irrespective of the current reputation profile. Therefore, optimally a good reputation is maintained or any reputation building process is completely neglected. However, our experimental results show that not all of the subjects follow the optimal strategy in the according treatment. Rather, the propensity to choose high quality is higher the more accurate customers are able to rate. Further, the chosen qualities are conditional on the current reputation profile. In particular, we see that subjects use milking strategies and exploit a good reputation. More precisely, if the sales price is high, low quality is delivered until the price drops below a certain threshold as a result of negative ratings, and then high quality is chosen until the price has significantly increased.

Our work directly contributes to the theoretical and empirical literature on reputation systems in online markets. The effect of repeated interaction on quality choices and the influence of a reputation system has been intensively studied in the relevant theoretic literature and is, e.g., surveyed in [3] or in [1] for online markets. Our theoretical model makes several modifications compared to the existing game-theoretic approaches. Most closely related to our setting is the model described in [4], which focuses on reputation systems for Internet transactions between a long-lived seller and short-lived buyers. The focus in [4] lies particularly in efficiency considerations if the length of the reputation profile or the number of negative ratings changes. From a game-theoretic perspective, in [4] perfect public equilibria (see [5]) of an appropriate repeated game with infinite horizon are analyzed. Also in [6] a model is investigated with a long-lived seller and short-lived buyers. In that particular model, quality is drawn randomly at the beginning of each period and the focus of the analysis is on the seller’s strategic decisions to honestly communicate the observed quality. [7] is interested in the short-lived buyers’ incentives to acquire costly reputation information about the long-lived seller’s past actions while the seller decides on the product quality.

Our work is also connected to the stream of empirical field studies and experimental research of reputation systems in online markets. For instance, [8], [9], [10], [11], [12] and [13] use the online market platform eBay to analyze different aspects of reputation in a field setting such as reputational effects on sales prices and the impact of negative/positive feedback on the number of sales and exit decisions. In the experimental study of [8], one finding is that more established sellers are able to sell their products at higher prices. While the effect of unfair negative ratings turned out to be not significant in [8], other empirical studies such as [11] and [12] observe a dependence of the seller’s price on the current reputation profile. A further consequence of negative ratings includes an increased probability that sellers with lower reputation are forced to leave the market [12]. The study of [13] shows that consumers are more willing to pay higher prices if sellers direct a fraction of the winning bid to a charity of their choice. In addition, eBay sellers who tie their products to charity donation increase on average the probability of a sale and receive lower complaints if they deliver lower quality than promised. Especially new eBay sellers who do not have a sales record yet, can credibly signal trust by using the option of charity donation. A detailed overview of different studies on the reputation system of eBay can be found in [8] (cf. Table 1) and [14] (cf. Table 1). Different studies, such as [15], [16], [17], [18] and [19], use a (modified) trust game within a laboratory experiment to analyze the effects of a reputation system on strategic decisions of buyers and sellers. A common finding is that reputation systems increase trust and trustworthiness between buyers and sellers. Buyers, whose trust was betrayed in one trade, are more reluctant to trust the sellers in the future, whereas positive experience maintains the level of trust for the next trade. In the random stranger matching treatment reported in [20] sellers who were trustworthy in the first phase of the according trust game, exploit in the second phase their good reputation by returning significantly less than before. The fact that the seller’s history gets not updated anymore generates incentives for the seller to exploit the buyer’s trust. Similar evidence of exploiting or milking one’s good reputation concerning future behavior can be observed in [21]. In a two-stage repeated trust game when there is disclosure of behavior to others in the first stage, sellers strive to reach high reputation by responding trust. However, without disclosure in the second stage and when buyers have to rely on the disclosure of the first stage, especially sellers who were very trustworthy in the first stage exploit their good reputation by returning significantly less in the second stage than before. [22] identify one seller strategy as best response to first return the trust of buyers consecutive times and then constantly exploit the trustworthy reputation by returning nothing anymore. Different to these common repeated trust games, in our setting milking strategies are expected even if the reputation system is still available and the reputation profile of the seller updated. As will be outlined below the strategies are more sophisticated and diverse than in common repeated trust games. Lastly, in our setting cycles of milking behavior are possible so that sellers can build and exploit good reputation multiple times which is different to milking in repeated trust games where once established high reputation is then constantly being exploited without re-establishing high reputation through costly trustworthy behavior again. Another interesting observation in [17] is that increasing the noise of the reputation system has the effect that buyers are less likely to react negatively to poor reputation profiles of the seller. Moreover, according to the experimental results in [19] there is a self-adjustment of the market to unfair negative ratings and, therefore, there is no influence of these ratings on the quality decisions of the seller compared to fair ratings. To the best of our knowledge, our study is the first in this experimental literature which models the customer exogenously and non-strategically. Our experimental design thus eliminates effects that may be induced by strategic rating behavior or reciprocity and enables us to identify causal effects of the reputation system on the quality choices of the service provider.

**Table 1:** Treatments.

$\bar{P}=60$, *γ* = 5 *c*_*H*_ = 50, *c*_*L*_ = 35	T1	T2	T3	T4
*α*	0.95	0.95	0.70	0.70
*β*	0.05	0.30	0.05	0.30
optimal strategy	*H*	*H*	*H*	*L*

## Theoretical model

We consider one long-lived service provider (seller) who repeatedly sells a service (or product) to short-lived customers. The seller has the strategic option to deliver the service in either high or low quality (at higher or lower cost, respectively). We assume that the service is an experience good, so that the true quality cannot be observed by the customer prior to purchase (and consumption). As a consequence, (online) evaluation systems aim at bridging the information gap, asking the customer to give a positive or negative quality rating after trade.

The service provider’s repeated decision problem is modeled as a (stationary) Markovian decision process (MDP) in discrete time with infinite horizon as in [23]. While a precise discussion of the MDP is given at the end of this section, we describe the basic ingredients here. The process runs over time, denoted by *t* = 0, 1, 2, …, and moves from state to state. A state is a vector of customer ratings of the *r* = 3 most recent periods. In principle, one may think of periods as quarters, months, days or single transactions. In each period customers purchase and rate the service. A single customer’s rating is either positive or negative. Based on the customers’ evaluations, an overall positive rating denoted by “+”, or an overall negative rating, denoted by “−” for the underlying period is derived. For expositional reasons, we assume that in each period a single transaction takes place with a customer’s rating of either “+” or “−”. Consequently, a state is a vector of the three most recent customer evaluations and the eight possible states are (+++), (++−), (+−+), (+−−), (−++), (−+−), (−−+), and (− − −) (newest rating on the right). States are also termed reputation profiles.

**Fig 1:**
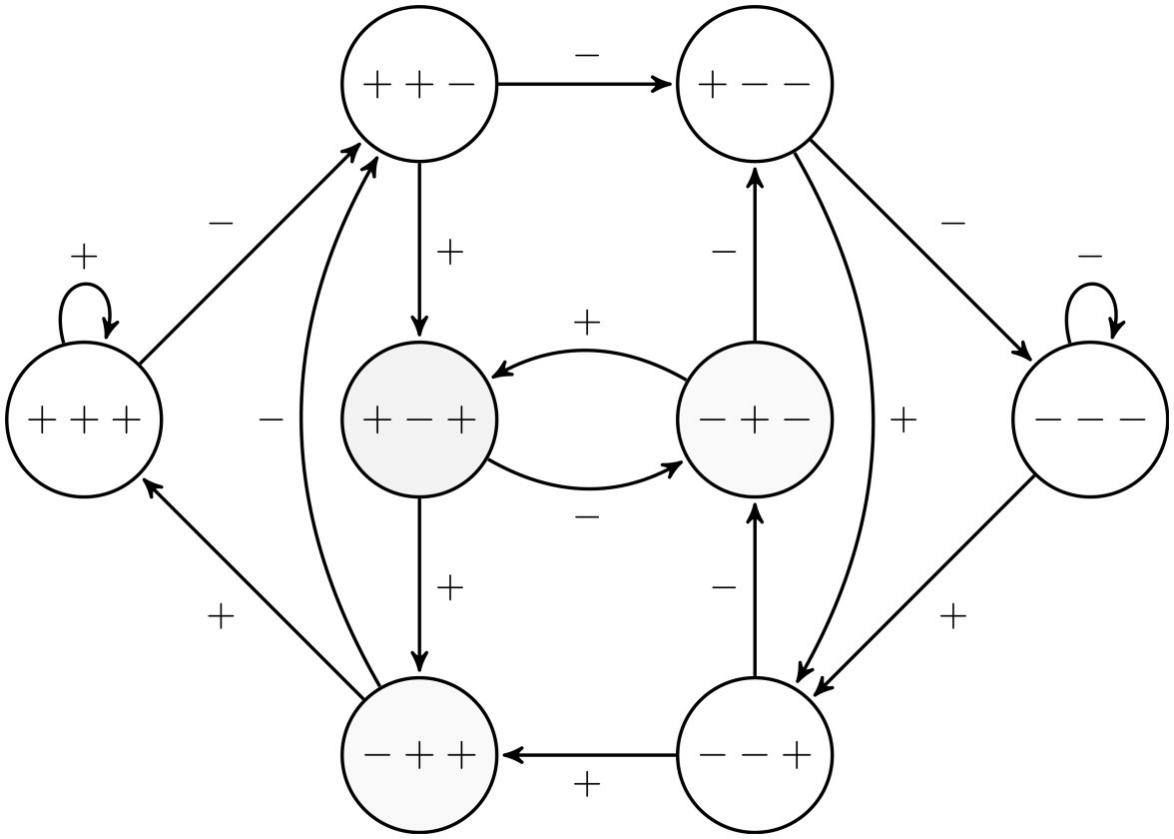
States and transitions for a reputation profile with 3 ratings.

These states along with the feasible transitions represented by the arrows for a reputation profile with three ratings are shown in Fig 1. As an example the reputation profile (+ − +) might change to the state (− + +) or (− + −), depending on whether the new evaluation is positive or negative, respectively.

Transitions to the next state are driven by the service provider’s quality decision. At each time *t* the service provider decides on either producing a service in high (*H*) or low (*L*) quality at a cost of *c*_*H*_ or *c*_*L*_, respectively. We assume *c*_*H*_ > *c*_*L*_. After delivery of the service, the service provider is rated by the customer. Since we focus on the provider’s quality decisions, we model ratings to be stochastic, i.e., they are subject to errors.

More precisely, a positive rating for a high-quality service occurs with probability *α* ∈ (0, 1) and for a low-quality service with probability *β* ∈ (0, 1). It is natural to assume *α* > *β*. The probabilities *α* and *β* reflect the accuracy of ratings. The lower *α* is, the more inaccurate is the rating information on high-quality services, while a higher *β* increases inaccuracy for low-quality services. In the restaurant example, a given percentage *β* of customers may not be able to detect poor quality and give a positive rating instead, while only a percentage of *α* is able to detect good quality and rate it accordingly. The values of *α* and *β* might stem from the seller’s experience. It is also intuitive that the design of the rating system has an impact on its accuracy. For example, control questions can unveil a customer’s rating ability. In effect, the more accurate ratings are, the higher the value of *α* and the lower the value of *β*.

To illustrate transitions between states consider Fig 1 again and assume the current reputation profile is (+ − +). If the service provider chooses high quality, then the next state will be (− + +) with probability *α* and (− + −) with probability 1 − *α*. If he chooses low quality, then these states are reached with probabilities *β* and 1 − *β* respectively.

Positive ratings should have a positive effect on the seller’s profit. There are generally two ways to assure this. One can either argue that higher ratings trigger a higher demand, so that profits increase. Or, equivalently, a better reputation profile yields a higher willingness to pay and hence a higher sales price. To keep the analysis tractable and the experiment instructions accessible, we choose the latter. The relationship between the willingness to pay and the reputation profile is also supported by empirical evidence on wine markets [24] and of transactions in eBay auctions [8, 14]. Equivalently, in the restaurant scenario, the owner may want to or need to adjust prices according to how he was rated in the latest restaurant guide.

In our model, the sales price *P* depends on the current state, i.e., on the previous three ratings. In state *s* the sales price *P*(*s*) is given by $P\left(s\right)=\bar{P}+\gamma \sigma \left(s\right)$, where $\bar{P}$ is a fixed base price, *σ*(*s*) is the difference of numbers of positive and negative ratings in *s* and *γ* is a price increment. In other words, in each period a new rating enters the rating profile, while the least recent rating drops out of the profile. The price raises (by 2*γ*) if a negative rating drops out and a positive rating enters. Similarly, a price decrease (by 2*γ*) is the result from a vanishing positive rating replaced by a new negative one. The relationship between the sales price and the reputation profile of a seller has been observed and used in the experimental and empirical literature on reputation such as [19] (cf. p. 419) and the references therein.

The service provider’s immediate reward (from sales) therefore depends on the current state *s* and chosen action *a* ∈ {*H*, *L*} and is given by *π*(*s*, *a*) = *P*(*s*) − *c*_*a*_. As assumed, the service provider is long-lived, he seeks to maximize the overall profit, i.e., the (infinite) sum of discounted immediate rewards. A pure Markovian strategy selects an action for each possible state. It is optimal, if application of the strategy maximizes the overall profit. A strategy is stationary, if it is independent of the time *t* and constant, if it is independent of state *s*. While the discount factor *δ* is assumed to be close to one in the experiment, we leave it variable in the theoretical analysis.

Within this model we show that either the constant stationary strategy “always producing high-quality services” or “always producing low-quality services” is an optimal strategy. The purpose of Proposition 1 is to quantify the dividing line between the two cases.

**Proposition 1** (Optimal Strategies). *For all specifications of the model there is an optimal strategy for the service provider that is stationary and independent of the reputation profile*.

*Precisely*, *delivering high quality in each period and each state is optimal if*

$$\begin{array}{c} 2\gamma \delta (\alpha -\beta )(1+\delta +\delta^{2})-(c_{H}-c_{L})\geq 0. \end{array}$$ (1)

*If the reverse inequality holds, it is optimal to always deliver low quality*.

Precise definitions of the MDP and as well as the proof of Proposition 1 can be found in the Supporting Information S1 Appendix. If it is optimal to always produce high-quality services according to the condition in Proposition 1, then a good reputation is maintained by the service provider. The proposition in particular says that at least one of the constant strategies is optimal. Which one is optimal rests on the parameters of the model. Observe that not the exact probabilities *α*, *β* but only their difference *α* − *β* = (1 − *β*) − (1 − *α*) matters. Recall that *α* − *β* is the difference of probabilities for positive evaluations.

Intuitively, if the probability for receiving a positive evaluation does not depend much on whether high or low quality is delivered (*α* − *β* is small), then (1) is less likely to be fulfilled, which in turn implies that low quality is optimal. As a consequence, the more accurate the evaluation (*α* close to 1, *β* close to 0), the larger the right hand side and the more likely it is that delivering high quality is optimal.

Similarly, in terms of costs only the additional costs for high quality enter the criterion in (1). Apparently, a smaller cost difference *c*_*H*_ − *c*_*L*_ as well as a higher price increment *γ* increase the right hand side and therefore the incentives to produce high quality. Finally, a high discount factor corresponds to a high valuation of future profits and therefore the service provider should be interested in keeping the sales price high by producing high quality.

A reformulation of condition (1) of Proposition 1 yields a lower bound for the expected profit margin, relating our analysis to the work in [4]. More precisely, the profit margin in our model can be written as

$$\begin{array}{c} \frac{\left(\bar{P}+3\gamma )-(\bar{P}-3\gamma \right)}{c_{H}-c_{L}}\geq \frac{3}{\left(\alpha -\beta )(\delta +\delta^{2}+\delta^{3}\right)}. \end{array}$$ (2)

[4] discusses a monopolistic long-lived seller who in each period chooses an unobservable effort for a service that is sold in a second price Vickrey auction to one out of *m* short-lived buyers. The buyers’ valuations are supposed to be independently and identically distributed. After the winning bidder receives the service, he observes its quality and provides a non-strategic feedback in form of a rating. An online reputation system is used to collect and publish the buyers’ ratings. The short-lived buyers use this information to form a belief about the seller’s strategic quality choices. The probability distribution of the seller’s effort levels depend on the current reputation profile. Even if the formal approach is apparently different, the condition for the production of high-quality services and reputation profiles consisting of the last three ratings is very similar to the one for the expected profit margin of an honest seller in [4] (cf. Proposition 3, Case 1). Therefore, condition (2) establishes that the lower bound on the profit margin for the production of high-quality services increases if the probability difference *α* − *β* from receiving a positive rating increases.

For reputation profiles consisting of the last three ratings there are 256 stationary pure Markovian strategies in total. We know from the literature (see [25]) that an appealing strategy in our experiment might be to “milk” a good reputation. That means, whenever the actual reputation profile does not contain enough positive ratings, then high quality is produced to obtain more positive ratings and to drive up the price above a certain threshold. Afterwards, low-quality services are produced to exploit the high price until the price falls below the threshold again. Hereby, the whole profile consisting of the last three ratings or just the two last or the very recent rating can be taken into consideration.

This can be formalized as follows: Given a length *ℓ* with *ℓ* ∈ {1, 2, 3} and *k* ∈ {0, …, *ℓ* + 1}, a strategy is *k-responsive to negative ratings for length ℓ* if (a) quality *L* is produced for reputation profiles with less than *k* negative ratings among the *ℓ* most recent ratings and (b) quality *H* is produced in reputation profiles with exactly or more than *k* negative ratings among the *ℓ* most recent ratings.

**Fig 2:**
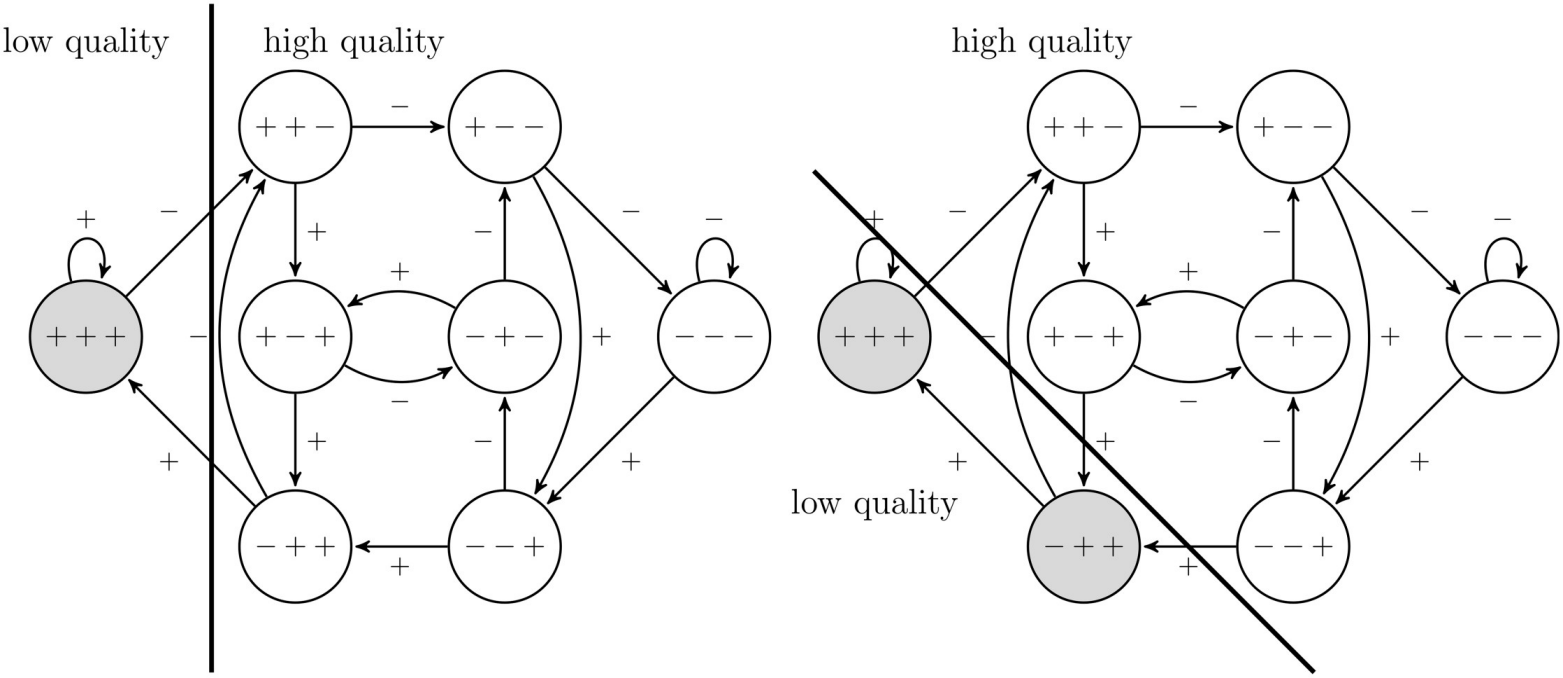
Examples for strategies that are 1-responsive for length 3 (left) and 1-responsive for length 2 (right).

Fig 2 shows two examples of *k*-responsive strategies. In Fig 2(left) the strategy is 1-responsive for length 3. This means a good reputation profile, having positive ratings in the last three positions, is exploited by producing low quality and for the remaining reputation profiles high quality is produced to improve the reputation profile and to drive up the price. In Fig 2(right) the strategy is 1-responsive for length 2, which means that the oldest rating is ignored and low quality is produced for reputation profiles with positive ratings in the two recent positions and high quality for all remaining reputation profiles. A detailed list of all *k*-responsive strategies as well as an illustration for *ℓ* ∈ {1, 2, 3} and *k* ∈ {0, …, *ℓ* + 1} can be found in the Supporting Information S1 Appendix, Table A and Fig A.

We close this section by remarking that the dependence of sales prices on the reputation profile is simple in the sense that it, e.g., does not account for an untainted reputation, so that there is a premium at state (+++). On the one hand, given that the optimal strategy is to always produce high quality, an additional premium at a top reputation will apparently not alter the optimal strategy. On the other hand, when always low quality is optimal, a sufficiently high premium may alter optimality to produce high quality and maximize the probability of being in best reputation.

Starting with [26], the literature on such premiums focuses on equilibrium pricing rules, i.e., on how prices should depend on (perceived) quality so that markets are cleared. In contrast to our setup, consumers are assumed to repeatedly purchase from the service provider. A “no-milking condition” is part of the equilibrium concept and requires that the service provider have no incentive to increase a one-period payoff by cheating and then leaving the market. In our model, which is the basis for the experiment described in the next section, the price mechanism is exogenous so that milking is not optimal. This allows us to study, whether subjects adhere to optimal behavior or deviate to milking.

## Experiment

This section introduces the experiment we conducted. We first describe the experimental design and the according procedure. Then, we develop our research hypotheses.

### Experimental design

Our experimental design is aimed at finding out, how service providers make their quality choices. The quality choices derived from our theoretical model serve as benchmark, so that we can compare observed quality choices with (calculated) optimal behavior.

**Fig 3:**
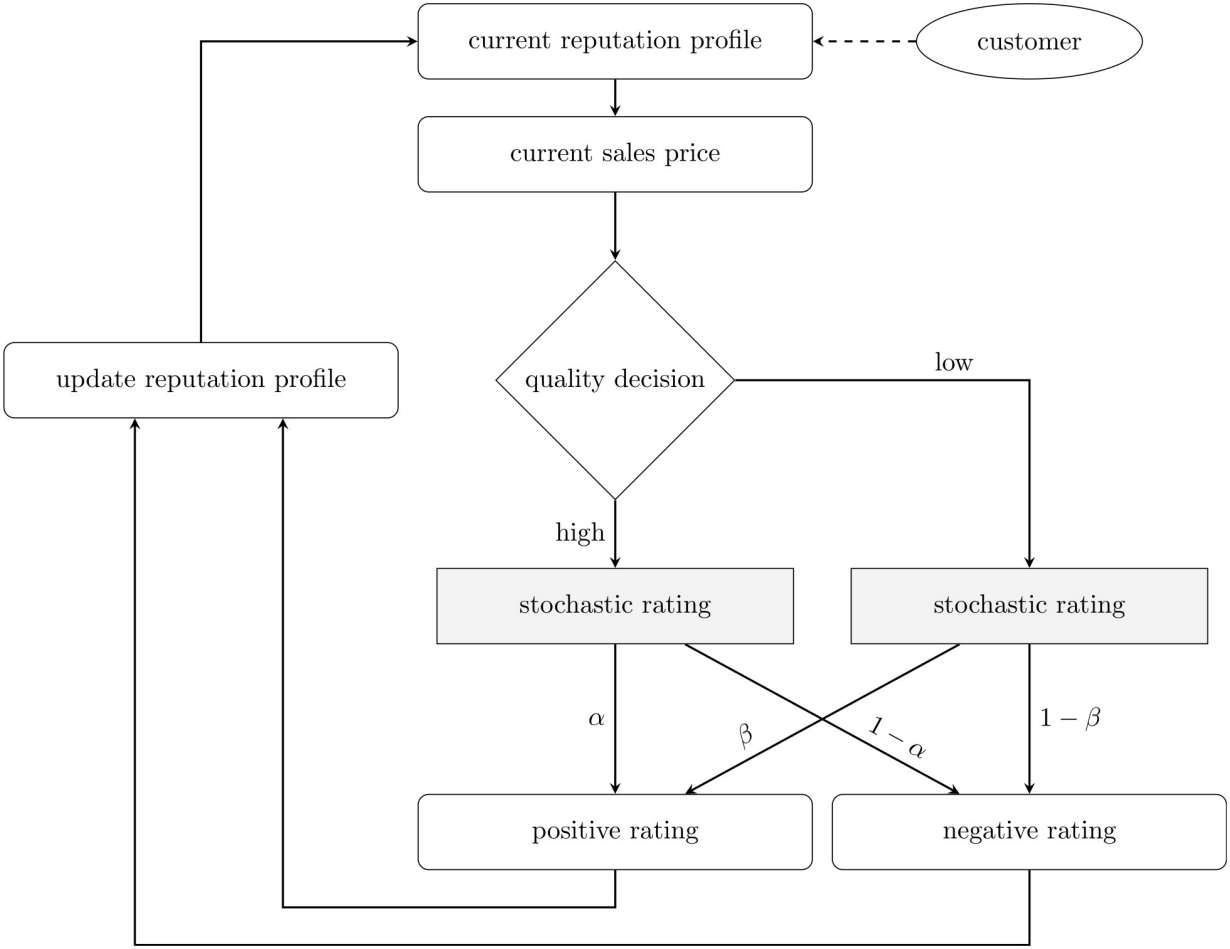
Experimental design: Subjects in the role of a service provider.

In our experiment, all subjects are in the role of a service provider. The customers are modeled exogenously and always demand a service of high quality. As discussed in the theoretical analysis, the price a customer is willing to pay depends on the service provider’s ratings of the previous three customers. The price the next customer is willing to pay increases with each additional positive rating of the service provider and decreases if the service provider received an additional negative rating from a previous customer. Thus, the higher the number of positive (negative) ratings, the higher (lower) the price the customer is willing to pay. For the sake of simplicity the price sensitivity is symmetric such that the willingness to pay increases and decreases with each change of rating at the same rate. At the beginning of the experiment, each service provider starts with a reputation profile of three ratings. We hold the starting conditions constant across subjects. Each service provider starts with the same most neutral reputation profile of two positive and one negative rating (+ − +). By using this neutral profile we avoid to send a signal on how the optimal strategy may look like, which would have been the case when the initial profile entirely contains either positive or negative ratings. The service provider decides in each of 40 consecutive periods between producing a high- or a low-quality service. In particular, each service provider encounters in each period the following choice problem: either producing a low-quality service which comes at low costs but probably negatively affects the price of the service in future periods, or producing a high-quality service which matches the customer’s preferences. A high-quality service probably leads to high prices for the service in future periods but also comes at high costs. After each decision the current customer gives either a positive or negative rating about the service. Congruent to the assumptions of the theoretical model, the customer in our experiment cannot evaluate the quality of the received service to the full extent and stochastically rates the quality of the service with predetermined probabilities *α* and *β*. The customer gives a positive rating in case of a high-quality service with a probability of *α* and in case of a low-quality service with a probability of *β*. We assume *α* > *β*. The rating determines the price the service provider will receive from the next period’s customer. The service provider’s reputation profile and the current sales price are updated after each decision accordingly. The decision process of the service provider in our experimental design is illustrated in Fig 3.

We develop four different treatments by varying the precision with which customers are able to rate the service provider positively or negatively when receiving a high- or low-quality service. More precisely, we alter the probabilities *α* of receiving a positive rating when supplying high quality between 95% and 70% and *β* of receiving a positive rating when supplying low quality between 5% and 30%. The parameters *α* and *β* in our four treatments are depicted in Table 1.

To clearly isolate the strategic quality choices of the service provider, we hold all other parameters of the theoretical model like the cost difference between high- and low-quality services, the price increment, and the service provider’s discount factor across all treatments constant. Note therefore that over all treatments there is a lower bound for the payoff from always choosing strategy *L* at 400 Taler (40 periods at a price of 45 Taler and costs of 35 Taler) and an upper bound at 1800 Taler (45 periods at a price of 75 Taler and costs of 35 Taler).

Eventually, we implement the infinite time horizon inherent in the theoretical model by the following standardized procedure (see for example [27, 28]): Period 1 to period 40 are played by each service provider. After period 40 a roll of a fair die determines, whether the service provider plays another period or not. For example, in the case that the six-sided die displays the numbers 1, 2 or 3 the service provider starts a new period, while in the case of a 4, 5 or 6 the experiment ends. We limit this procedure to a maximum of 5 additional periods, so that each service provider plays at least 40 periods and at most 45 periods. By extending the number of periods in this way, we avoid so-called “endgame-effects”. Suppose for example we would not have implemented this procedure and service providers know for sure that period 40 will be the last round. Then it is optimal for each service provider to produce low-quality, as the subsequent rating of the customer does not affect prices in the last period. However, by accounting for endgame-effects there is a sufficiently high probability that the game continues. Consequently, service providers have incentives to keep up with their previous decisions ensuring that quality choices in later periods are valued as much as quality choices in the beginning of the experiment. Thus, this allows us to include all 40 quality choices in our analysis.

### Experimental design and theoretical model

The main insight from the theoretical analysis was to identify conditions under which the stationary strategies to always produce high or low quality, respectively, are optimal (Proposition 1). While the theoretical model uses an infinite horizon Markov decision problem, the experiment has to terminate after finitely many rounds. To implement an infinite horizon in the experimental setup, we use the standard technique of *random termination* as introduced in [29]. In our experiments subjects know the minimal (40) and maximal (45) number of rounds. Without that knowledge it would be impossible to control subjects expectations on how long the process will continue. However, knowing a maximal number of rounds may cause so-called end-game effects, meaning that subjects may exploit the fact that, e.g., producing low quality in the last round incurs lower cost, while there is no subsequent effect of a more likely negative rating. In the analysis, we only included decisions in the first 40 rounds. At the end of round 40 there was the first random draw determining whether or not the process proceeds or not. Comparing subjects actions within the first 35 rounds with their actions over all 40 rounds, we find no significant difference, so that end game effects may be neglected in the interpretation of our results.

A second difference between model and experiments concerns the initial profile of evaluations. In the theoretical model, we showed that there is a constant (profile-independent) stationary (time-independent) optimal strategy. Since we are not concerned with concrete final payoffs, there is no need to specify an initial profile. Phrased differently, the optimality condition holds for any possible initial profile. In the experiment, we have to choose an initial profile. For comparability of results all subjects need to start with the same profile (+ − +), which on the one hand is neither overly positive nor negative and thus place a bias on actions. On the other hand, it is supposed to remind subjects that ratings are stochastic. Moreover, the starting price associated with that profile leaves room to alter it in either direction through strategic choices.

Finally, the infinite horizon model requires a discount factor *δ* < 1 to guarantee finiteness of present values. In the experimental setup, we did not add discounting of future payoffs in order to keep the instructions and payoffs as simple as possible and because the time needed to conduct the experiment was short (about 30 minutes). The given optimal strategies (“always high” in **T1–T3** and “always low” in **T4**) refer to a discount factor close to 1. Inspection of the optimality condition (1) reveals that in all treatments the range of possible discount factors, for which the given optimal strategy is still optimal, is considerably large, demonstrating robustness of optimal decisions. More precisely, in **T1** (**T2** and **T3**) the optimal strategy remains optimal for *δ* > 0.73 (*δ* > 0.87). In **T4**, the optimal strategy is even optimal for any discount factor between 0 and 1.

### Experimental procedure

The experiment was conducted at the Business and Economic Research Laboratory (BaER-Lab) in June 2014 at the University of Paderborn, Germany. Subjects were recruited by the online recruiting system ORSEE [30] from a pool of approx. 2,200 voluntary students of the University of Paderborn from different fields of study, who are enrolled as prospective participants in economic experiments. We ran two sessions for each of the four treatments. In total, 206 subjects of various fields of study were distributed evenly across the sessions, resulting in 49 to 53 subjects per treatment. Each subject participated in one session only. The experiment was programmed and conducted with the software zTree [31]. As soon as the subjects arrived at the BaER-Lab, they were asked to randomly draw a number from a box and were told to sit down at the assigned computer workplace in a cubicle detached from each other ensuring complete anonymity. In each session, the subjects received the same introductory talk and were told not to communicate during the complete session. Then, the written instructions were handed out and the subjects had ten minutes time to read them and ask questions in private to clarify any misunderstandings. The detailed instructions for our experiment can be found in the Supporting Information S1 Appendix. In the instructions, subjects were assured that all decisions were made anonymously, so that neither the experimenter nor other participants got to know the identity of the subject who made a specific decision. Each subject only had to decide for him- or herself and was not informed about the decisions of others. Furthermore, the subjects were informed that their payoff was given to them anonymously and that their payoff did not depend on the decisions of the other subjects. At the end of the experiment, the subjects were asked to fill out a questionnaire which included questions about their socio-economic background such as age, gender, and field of study. Each session lasted for about one hour and subjects earned 12.50 Euro on average (roughly 16 USD at that time). Subjects were paid according to the final balance of their account at an exchange rate of 1 Euro per 100 Taler. In addition, all participants received a show-up fee of 2.50 Euro. The payoffs in Taler observed in the experiment as well as the potential payoffs subjects would have received by following the optimal strategy are summarized in Table 2.

**Table 2:** Observed payoffs in Taler for an initial account of 225 Taler.

	T1	T2	T3	T4	total
subjects	52	49	53	52	206
mean	1109.71	1126.43	831.51	935.10	998.03
standard deviation	78.64	70.51	82.80	93.36	147.95
minimum, maximum	950, 1250	965, 1250	665, 1120	770, 1115	665, 1250
optimal strategy	1316.34	1313.47	1126.23	1004.81	1190.21

### Research hypotheses

We develop the following research hypotheses based on theoretical predictions and behavioral conjectures.

We use the condition in Proposition 1 for the parameters chosen within the different treatments to derive the optimal strategy for the service provider. As shown in Table 1 this optimal strategy is to always produce high-quality services in **T1**, **T2**, and **T3**, i.e., to maintain a good reputation, whereas in **T4** producing low-quality services is optimal, i.e., completely refraining from any reputation building process. To be more precise, condition (1) in Proposition 1 shows that the comparative advantage of the optimal strategy depends on the difference of receiving a positive rating for high quality compared to the according probability for low quality. Thus, the exact probabilities should not influence the strategic quality choice of a service provider as long as their difference lies within a determined range. For example, in **T2** the difference between *α* and *β* is smaller compared to **T1**. However, as the difference still meets condition (1) the same frequency of producing high quality should be the same compared to **T1**. Thus, it follows from Proposition 1 that there should be no difference in choice behavior between treatments **T1**, **T2**, and **T3**. Whereas these model predictions about optimal behavior stem from rationality assumptions, research in behavioral economics -a field of economics which seeks to increase the explanatory power of economics by providing it with more realistic psychological foundations- provided ample evidence that humans have cognitive biases in correctly judging the likelihood of events and make decisions that violate the standard principles of probability calculus (for an overview see [32] or [33]). One of these theories is the prospect theory discussed in [34], which suggests that people discard events of extremely low probability and treat events of extremely high probability as if they were certain. Considering **T1** the high value of *α* of 0.95 and likewise the low value of *β* of 0.05 will lead subjects to evaluate the event of receiving a positive rating when producing high quality as almost certain and the event of receiving a positive rating when producing low quality as almost impossible. However, according to the theory, decision-makers are sensitive to probability changes especially when turning away from either extremely high or extremely low probabilities in absolute terms, so that these risky events will be less over- or underestimated. Consequently, changing the absolute value of receiving a positive rating when producing either low or high quality will thus lead to different choice behavior in **T2** and **T3** compared to **T1**. Particularly, compared to **T1**, the option to produce low-quality seemingly becomes more attractive by raising *β* in **T2**, while decreasing *α* by the same amount seemingly lowers the attractiveness of producing high-quality in **T3**. Thus, from a behavioral perspective, we expect that the choice behavior will differ across the treatments and more deviations from optimal behavior will be observed the lower the difference between *α* and *β* becomes. Accordingly, we test the following hypothesis:

**Hypothesis 1**. *The frequency of choosing high quality decreases as the difference between α and β decreases*.

Even if online markets employ reputation systems, it is not uncommon to observe oscillations in quality [35]. For example, service providers might periodically choose actions that deviate from their usual (optimal) strategy, which is even more likely to be observed if customers may make mistakes in evaluating the quality of the service correctly. Hence, besides the optimal strategy, a natural and plausible candidate would be to exploit the positive reputation profile and the associated high price to provide low quality for a short period of time.

This means that up to a certain threshold, negative ratings are acceptable and low-quality services are produced to profit from high prices. If then the price drops below this threshold, high-quality services are produced again to induce a price increase. This strategy entails that the service providers react to the ratings they receive. We assume hereby that the observed actions are still pure Markovian but vary over states of possible reputation profiles. Thus, whenever the subjects face the same history of the last previous three ratings, they choose the same action. We denote this form of strategy that reacts to negative ratings as a *milking strategy*. Formally, we call a strategy a *milking strategy* if it is *k*-responsive to negative ratings for length *ℓ* with *k* ∈ {1, …, *ℓ*} and *ℓ* ∈ {1, 2, 3}. Compared to the former definition of *k*-responsiveness we excluded the two state independent-strategies with always high quality (*k* = 0) and always low quality (*k* = *ℓ* + 1) to be a milking strategy. Already [25] (cf. p. 29) posed the question if such an oscillating strategy “to repeatedly building up a reputation and then milking it” can describe the optimal quality choice over time. That paper demonstrates in a continuous time model that under technical assumptions, such as concavity in the control variables, firms optimally choose a steady-state quality level [25] (cf. Theorem 4). This is directly in line with our theoretical findings. Also [4] (cf. Section 3.4, p. 218) refers to these types of milking strategies and investigates how they can be avoided in the context of costless identity changes. Our conjecture is that when a service provider repeatedly chooses the quality of his services, we will indeed observe in an experimental setting that subjects make use of milking strategies even if these strategies are not optimal according to Proposition 1. This is our second hypothesis.

**Hypothesis 2**. *Subjects use a non-optimal milking strategy*.

## Experimental results

The empirical analysis of our experimental observations starts with descriptive statistics. Then, we present the results of nonparametric tests based on individual quality choices to investigate our first research hypothesis. To investigate the second hypothesis on whether subjects employed milking strategies we first compute the nonparametric Brier score to identify which milking strategies were used and, subsequently, use multivariate regression analysis to corroborate our findings. The empirical analysis has been conducted with *Stata 13.0*.

### Descriptive statistics

Table 3 summarizes the individual choice behavior across each single treatment. The absolute number of periods a particular reputation profile was obtained and the relative frequency of choosing high-quality service *H* in each reputation profile are displayed. Hereby in Table 3, the average relative frequency means that for each reputation profile and for each subject individual choices for the high-quality service are averaged across periods and then averaged across subjects. As can be seen in Table 3, subjects numerically chose on average the high-quality more often than the low-quality service in all treatments and for all reputation profiles. In addition, choosing high-quality in treatment **T1** is numerically more pronounced in all possible reputation profiles compared to the treatments **T2** and **T3** which all propose to pursue the optimal strategy of always choosing the high-quality service. Interestingly, although the average number of obtained reputation profiles with three and two positive ratings looks very similar across the treatments **T1** and **T2**, the average relative frequency of choosing high quality differ constantly throughout each possible reputation profile. This tentatively implies that subjects in **T2** obtained these reputation profiles by choosing the low-quality service more often, hoping for a positive rating because of the higher *β* and thus, the increased inability of the customer to correctly evaluate the low-quality service. Comparing the frequency of the high-quality alternative between **T1** and **T3**, subjects in **T3** numerically also chose high quality less often, but in contrast to **T2**, these subjects less often obtained reputation profiles of three or two positive ratings because of the lower *β* (which is 0.05 compared to **T2** with a value of 0.30). Lastly, the average frequency of choosing the high-quality service among all treatments is lowest in **T4**; however, the subjects in this treatment chose this option numerically more frequently than low quality. To test to what extent the observed behavior in the treatments deviate from optimal behavior we count how many times the high-quality service was chosen for each subject in the treatments **T1**, **T2** and **T3** and compare these numbers with hypothetical subjects who would have chosen the high-quality service in all 40 periods. For subjects in **T4** we compare their choice behavior with hypothetical subjects who would have chosen the low-quality service in all 40 periods. Finding no significant difference would support the view that the subjects in the according treatments indeed played the optimal strategy on average. The results of the Mann Whitney U-Test, however, suggest that in all treatments the actual frequency of optimal choices are statistically different from the expected frequency of optimal choices (*Mann Whitney U-Test*, each subject one independent observation, **T1**: *z* = 7.309, *p* < 0.0001; **T2**: *z* = 8.395, *p* < 0.0001; **T3**: *z* = 9.209, *p* < 0.0001; **T4**: *z* = −9.209, *p* < 0.0001). This observed choice behavior clearly implies that deviations from optimal behavior are not due to smaller mistakes. Rather, on average subjects do not constantly choose the high-quality or the low-quality alternative, although this would have been optimal. Thus, observed behavior is significantly different from optimal behavior, which supports our behavioral conjecture that a significant proportion of subjects uses strategies different from the optimal ones proposed by our theoretical model.

**Table 3:** Choice behavior across reputation profiles and treatments.

	T1		T2		T2		T4	
reputation profile	frequency of obtained reputation profiles (left) and average relative frequency for *H* (right)							
(+ + +)	1040	0.791	803	0.674	276	0.749	339	0.541
(+ + −)	270	0.865	247	0.810	286	0.822	275	0.678
(+ − +)	293	0.917	298	0.771	306	0.818	324	0.694
(− + +)	302	0.863	270	0.768	297	0.789	296	0.637
(+ − −)	61	0.937	95	0.829	232	0.754	216	0.742
(− + −)	41	0.875	104	0.789	210	0.743	225	0.660
(− − +)	57	0.944	87	0.864	215	0.881	204	0.799
(− − −)	16	0.809	56	0.801	298	0.680	201	0.670
	2080		1960		2120		2080	

### Comparison of theoretically proposed and observed quality choices (Hypothesis 1)

Recall from our theoretical model that the optimal strategy in all treatments **T1**, **T2** and **T3** is to choose in each period high quality *H* independently of the current reputation profile. Likewise, in treatment **T4** the optimal strategy is to choose in each period low quality *L* independently of the current reputation profile.

To investigate Hypothesis 1 we test the null hypothesis that observed quality choices do not depend on the customer’s evaluation abilities. We concentrate our analysis to **T1**, **T2** and **T3** as in all of these treatments the optimal strategy is to always produce high quality, irrespective of the accuracy of the ratings. Specifically, choice behavior across the treatments **T1**, **T2** and **T3** should be the same even if the difference between the probabilities *α* and *β* of correctly evaluating the service decreases from **T1** to **T2** and **T3**. We find that the frequency of high-quality choices in treatment **T1** is significantly greater than the corresponding frequencies observed in **T2** and **T3**, respectively (*Mann Whitney U-Test*, each subject one independent observation, **T1** vs. **T2**: *z* = 3.237, *p* = 0.0012; **T1** vs. **T3**: *z* = 3.579, *p* = 0.0003). Hence, high-quality choices are observed less if customers’ ratings are less accurate, either by lowering (raising) the probability *α* (*β*) that customers will give a positive rating for high quality (low quality). However, if the difference between *α* and *β* is held constant, we do not find a statistical difference in choices (*Mann Whitney U-Test*, each subject one independent observation, **T2** vs. **T3**: *z* = 0.0970, *p* = 0.9225). Thus, we can support Hypothesis 1 that the frequency of choosing high quality decreases as the difference between *α* and *β* decreases.

### The use of milking strategies (Hypothesis 2)

Referring to the findings of the previous sections, a significant proportion of subjects chooses strategies different than proposed by our theoretical model. With Hypothesis 2 we aim to gain further insights into this observed choice behavior. Before we start with the analysis of the subjects’ strategies we first ensure that the individual quality choices are consistent and, thus, in accordance with the assumptions of our theoretical model (i.e. the use of stationary pure Markovian actions). We call the individual quality choices of one subject consistent if the subject chooses on average for each given reputation profile the same quality of the service (i.e. when obtaining the reputation profile (+ − −) the subject always chooses *L*). Hence, for each reputation profile and each subject we look at the percentage of consistent actions and then average those over subjects. Table 4 shows that the subjects chose the same quality alternative in each reputation profile in 85% of all quality decisions on average. When taking a usual percentage of erratic decisions into account [36], we can interpret this choice behavior as consistent and consider the assumption of our theoretical model as met for the subsequent analysis.

**Table 4:** Consistent choice behavior across treatments.

reputation profile	(+++)	(++−)	(+−+)	(−++)	(+−−)	(−+−)	(−−+)	(− − −)
number of observations	198	202	206	205	167	152	162	118
average consistent actions	0.860	0.857	0.851	0.858	0.863	0.827	0.887	0.833
standard deviation	0.161	0.167	0.176	0.166	0.173	0.194	0.174	0.177

Next, we analyze the subjects’ strategy choices. We partition the space of strategies into optimal and milking strategies and investigate to what extent subjects follow one of these strategies. A detailed list of these strategies can be found in the Supporting Information S1 Appendix, Table A. To determine which of these strategies a subject most likely followed we compare the quality alternatives a subject actually chose with those that should have been chosen according to the strategy under consideration. More precisely, we look at every period and assign one point if the subject acted according to this strategy and zero points otherwise. We sum up these points over all periods and obtain a score between 0 and 40 that indicates the similarity between the subject’s choice behavior and the strategy under consideration. We repeat this scoring rule for all optimal and milking strategies and assign to each subject the strategy with the highest score. Hereby, if the scores of the most likely chosen strategies for one subject are too close to each other (within a range of 3 points), we exclude this subject from the sample. Overall, we had to exclude 42 of the 206 subjects because the high scores of at least two strategies were within a range of 3 points. To verify that our scoring rule correctly identified the strategies of the remaining subjects, we compute the so-called Brier score which is commonly used as verification measure for assessing the accuracy of probability forecasts [37, 38]. The essential idea behind the Brier score is that it computes the average gap (mean squared difference) between the forecast and the actual outcomes. The lower the gap between predictions and outcomes, the more accurate are the probability forecasts. Further details on the Brier score can be found in [39] and [40]. The Brier score is also used in economic experiments for matching observed decisions with game-theoretic predictions (see for instance [41, 42]). In our analysis the Brier score compares the observed quality choices with the subject’s assigned strategy and gives a measure of how accurate the quality choices match the strategy prediction. If, for example, the milking strategy of 1-resp 2 predicts that subjects are expected to choose the low-quality service in the states (+ + +) and (− + +) and to choose the high-quality service in any other state. We can relate these forecasts with the actual choices of precisely those subjects who we identified to play most likely this according strategy of 1-resp 2. As implemented in *Stata 13.0*, a perfect forecast yields a score of 0.00, while a perfect mismatch in forecasting gives a score of 1.00. Thus, the lower the Brier score the better the fit, which means that the specific subjects under consideration most likely followed the assigned strategy. Table 5 shows the distribution of optimal and milking strategies used by the subjects and the corresponding Brier scores.

**Table 5:** Distribution of optimal and milking strategies in the experiment.

	optimal strategies		milking strategies					
	*H*	*L*	*k*-resp *ℓ* with 0 < *k* < *ℓ* + 1 for length *ℓ* = 1, 2, 3					
	0-resp 3	4-resp 3	1-resp 1	1-resp 2	1-resp 3	2-resp 2	2-resp 3	total
**T1**	35	0	0	6	8	0	0	49
	71.43	0.00	0.00	12.24	16.33	0.00	0.00	100.00
	33.98	0.00	0.00	40.00	38.10	0.00	0.00	25.24
**T2**	27	6	4	2	3	1	0	43
	62.79	13.95	9.30	4.65	6.98	2.33	0.00	100.00
	26.21	42.86	66.67	13.33	14.29	50.00	0.00	26.22
**T3**	23	3	1	1	6	1	2	37
	62.16	8.11	2.70	2.70	16.22	2.70	5.41	100.00
	22.33	21.43	16.67	6.67	28.57	50.00	66.67	22.56
**T4**	18	5	1	6	4	0	1	35
	51.43	14.29	2.86	17.14	11.43	0.00	2.86	100.00
	17.48	35.71	16.67	40.00	19.05	0.00	33.33	21.34
Brier score	**0.1393**	**0.2240**	**0.2750**	**0.1350**	**0.1140**	**0.1500**	**0.3167**	
total	103	14	6	15	21	2	3	164
	62.80	8.54	3.66	9.15	12.80	1.22	1.83	100.00
	100.00	100.00	100.00	100.00	100.00	100.00	100.00	100.00

*Note.* The first number in each cell is the number of subjects that followed the strategy under consideration. The second number is the percentage of this strategy compared to all strategies among the subjects for a particular treatment and the third number is the percentage of this strategy over all treatments **T1** to **T4**.

The first number in each cell is the absolute number of subjects that followed the strategy under consideration in a particular treatment. The second number highlights in percentage how often this particular strategy was used compared to all other strategies we identified among the subjects for a particular treatment. Lastly, the third number indicates in percentage how often a particular strategy was used over all treatments **T1** to **T4**. For instance, in treatment **T1** in total 35 subjects followed the optimal strategy of always choosing high quality (0-resp 3). Compared to all other strategies observed in **T1**, this strategy has been used with a frequency of 71.43% and with a frequency of 33.98% across all treatments **T1**-**T4**. This strategy of always choosing high quality, which is optimal for treatments **T1**, **T2** and **T3**, has been pursued by 103 out of 164 subjects. This is more than 60% of the subjects. Even if this was not optimal in treatment **T4**, still 18 out of in total 52 subjects have decided to follow this strategy. Note that there has been no subject following a strategy that is 3-responsive of length 3 (i.e., high quality is only chosen, when the reputation profile is (− − −)). Still, we find compelling evidence that parts of our subject sample indeed used milking strategies. In most of the cases subjects have chosen to follow a milking strategy that is 1-responsive (i.e., 1-resp 1, 1-resp 2, 1-resp 3 which is 42 out of 164 subjects). This means that the price was driven up until the reputation profile consisted of positive ratings in the last two or three positions. Then, the high price was exploited by choosing low quality and driven up by choosing high quality again as soon as the subjects received a negative rating. Note that subjects actually earn less if they pursue a milking strategy instead of following the optimal strategy. Given the experimental parameters, subjects who used strategies of 1-responsive length 1, 2 and 3 earned on average 999.17 Taler, 984.67 Taler and 988.81 Taler, respectively. Subjects who used strategies of 2-responsive length 2 and 3 earned on average 850.00 Taler and 861.67 Taler, respectively. Referring to Table 2, subjects would have earned 1190.21 Taler if they had followed the optimal strategy, thus losing on average roughly 220 Taler. Looking at the Brier scores we observe that the assigned strategy predicts subject’s behavior well for the optimal strategy *H* and the milking strategies that are 1-responsive of length 2 and 3. The behavior of the milking strategy that is 2-responsive of length 2 is also predicted fairly well; however, as in the case of strategies of 1-responsive length 1 and 2-responsive length 3, the rather low number of observations warrants some caution in interpreting the results. Overall, with respect to Hypothesis 2 we observe that according to the Brier score milking strategies are indeed used by 47 out of 164 subjects who pursued an unambiguous strategy. This proportion is significantly higher than 0 (*Proportional z-Test*: *z* = 7.407, *p* < 0.0001), supporting Hypothesis 2 that parts of the subject sample use milking strategies.

We use a binary probability model to corroborate the presence of milking strategies. In our logistic regressions, the dependent variable is whether high quality was chosen (= 1) or not (= 0). We specify each milking strategy which was identified by our scoring rule as a binary independent variable. The reference group for each included milking strategy is the set of reputation profiles with an expected high-quality choice. For example, to run the regression on the strategy 1-responsive of length 2 the reputation profiles with expected low quality (+ + +) and (− + +) were coded to 1 and all other profile states to 0. Note that we use a panel estimator to run our regressions which clusters standard errors on the subject level. By doing this the regression model errors are independent across clusters but correlated within clusters, allowing us to include all single observations in our regression analysis. Regressions are run separately for the set of treatments (**T1**,**T2**,**T3**) with **T1** as the reference group and for **T4** only, due to different model propositions for the optimal strategy. For the sake of completeness we run the regressions for the complete sample and for the sub-samples which—according to our scoring rule—pursue the reported milking strategy. The regression results of the sub-sample can be found in the Supporting Information S1 Appendix, Table B. Because each of the 40 periods is one unit of analysis, a random-effects model is specified to account for unobserved individual-specific correlated errors.

Table 6 reports the odds ratios while robust standard errors are mentioned in parentheses.

**Table 6:** Results of logistic regression for presence of milking strategies (complete subject sample).

dependent variable: high quality	T1,T2,T3					T4				
	(1)	(2)	(3)	(4)	(5)	(1)	(2)	(3)	(4)	(5)
1-resp 1	0.787* (0.106)					0.850 (0.143)				
1-resp 2		0.370*** (0.061)					0.456*** (0.092)			
1-resp 3			0.287*** (0.058)					0.335*** (0.078)		
2-resp 2				1.107 (0.218)					0.886 (0.169)	
2-resp 3					0.711** (0.112)					0.589*** (0.110)
**T2**	0.286*** (0.106)	0.249*** (0.105)	0.240*** (0.108)	0.296*** (0.107)	0.280*** (0.106)					
**T3**	0.284*** (0.087)	0.200*** (0.070)	0.175*** (0.066)	0.316*** (0.095)	0.267*** (0.086)					
*period*	0.988*** (0.003)	0.988*** (0.004)	0.990*** (0.004)	0.989*** (0.003)	0.943*** (0.000)	0.986*** (0.004)	0.986*** (0.005)	0.987*** (0.005)	0.986*** (0.004)	0.984*** (0.004)
subjects	154	154	154	154	154	52	52	52	52	52
observations	6160	6160	6160	6160	6160	2080	2080	2080	2080	2080

*Note.* Odds ratios were calculated, robust standard errors are reported in parentheses. Each model specification (1)-(5) includes one milking strategy. Regression analysis for **T1-T3** is separated from **T4** due to different model propositions.**T1** is the reference group for **T2** and **T3**. Significance at the 1%, 5%, and 10% level is denoted by ***, ** and *, respectively.

Referring to Table 6 we see results similar to the non-parametric findings. With the exception of the insignificant estimate for the *2-resp 2* milking strategy, all estimates have the expected magnitude below 1. To be more specific, the number in each of the first five rows from 1-resp 1 down to 2-resp 3 displays the odds ratio of high-quality over low-quality choices for each strategy. For example, in the row 1-resp 2 the odds ratio of 0.370 means that in states in which the strategy predicts low-quality (which are the states (+ + +) and (− + +)) the odds of observing high-quality is roughly three times lower than the odds of observing low-quality. Phrased differently, the odds of observing low-quality choices in these states are three times as large as the odds of observing high-quality choices. By observing significant odds ratios below 1 thus means that high-quality is significantly less chosen in reputation profiles in which the according milking strategy also predicts low-quality.

Congruent to the Brier scores, this behavioral pattern is highly significant for 1-responsive milking strategies of length 2 and 3. Also in line with findings of the previous sections the probability of choosing high quality is significantly lower in **T2** and **T3**, compared to **T1**. This can also be seen from the significant odds ratios below 1. For example, the odds ratio of 0.249 in the cell of specification (2) and row **T2** means that high-quality was chosen four times less compared to **T1** given that in both treatments the milking strategy of 1-resp 2 was used. In addition, the *period* variable highlights that subjects behaved consistently as the significant odds ratios are near 1, meaning that the probability of choosing high quality does not depend on the periods and thus on the timing of the experiment. Taking together the findings derived from the non-parametric analysis and the multivariate statistics, we see Hypothesis 2 supported.

## Conclusion and future directions

In this study we examine how the reputation of a service provider affects his strategic behavior if customers cannot fully evaluate the quality of the delivered service. Our research strategy comprises two parts. First, depending on the customers’ evaluation abilities, we theoretically analyze the impact of reputation on the strategic quality choices of the service provider. We model the service provider’s profit maximization problem as a Markovian decision problem and derive the optimal strategy for reputation profiles consisting of the previous three ratings. A profit-maximizing service provider is expected to either always produce high-quality services or to always produce low-quality services. Therefore, we design situations in which the service provider optimally either maintains a good reputation or completely refrains from any reputation building process. We determine a condition that allows us to predict this optimal strategy depending on exogenously given parameters such as the customers’ evaluation abilities, the cost difference between high and low service quality, the price increment, and the service provider’s discount factor. Second, we design a laboratory experiment that is captured by our theoretical model. We are interested how subjects in the role of service providers make their quality choices and compare observed with optimal behavior. In particular, the assumption of an exogenous price mechanism allows us to study whether subjects adhere to optimal behavior or deviate to milking behavior, without any confounding effects from strategic reactions by the seller induced, for example, by price premiums for an untainted reputation profile. In the experiment we observe that not all of the subjects follow the optimal strategy. Further, the frequency of choosing high quality decreases as the difference between the probabilities of receiving a positive rating for high and low quality decreases.

Continuing the focus on the individual quality choices we reveal that a significant proportion of roughly one-third of the subjects uses milking strategies. In most cases, the price was driven up until the two or three most recent ratings in the reputation profile were positive. Then, the high price was exploited by choosing low quality and driven up by choosing high quality again as soon as the subjects received a negative rating. These results are corroborated by multivariate analysis.

Insights about the service provider’s behavior are in particular of practical relevance in anonymous settings, such as markets of experience goods, which are characterized by profound information asymmetries between customers and service providers. The use of milking strategies shows that from a behavioral perspective there is a tendency to exploit the customers by producing low-quality services if the reputation is sufficiently high and the customers’ evaluation abilities are sufficiently precise. However, from a theoretical perspective this strategy is not profit-maximizing. Thus, a managerial implication with respect to our findings is that firms should be aware that short-run gains from producing low-quality services lead to an increased probability of receiving a negative rating. This negative rating stays within the reputation profile for the next periods, influencing the next customers’ willingnesses to pay and therefore having a negative impact on future profits. Using a milking strategy, even if it is not optimal from a theoretical point of view, demonstrates that this effect is underestimated by the subjects acting as a service provider. In addition, there is also a tendency to follow the customers’ demand and to produce high-quality services even if this is not rewarded by receiving a positive rating with a sufficiently high probability. The subjects seem to be too cautious about actually choosing the profit-maximizing strategy and thus, we observe a too large share of high-quality services. This behavior may be due to the set of *α* and *β* probabilities which might not been salient enough to induce the optimal strategy of always choosing low quality. Another possible answer is that the behavior was driven by ethical concerns. Referring, for example, to the experimental literature about lying and cheating in economic situations, the majority of subjects deceive others, but only to a small extent [43–45]. Applied to our setting, subjects might have been ethically inclined to match the customer’s need in at least some periods irrespective of the economic gains and losses. The use of non-optimal milking strategies in the lab might also be explained from beliefs that subjects based on their experiences in online transactions outside the lab. In fact, the deterministic nature of the price formation process in combination with a short-lived customer makes it tempting for service providers to use a milking strategy. Outside the lab the danger of losing the reputation in the long run after delivering low quality at a high price might deter such a behavior. Knowing that a lasting loss of reputation is excluded by design in the experiment might lure subjects in milking their good reputation. The fact that this study is conducted in the laboratory with students as subjects sets some limitations to our work and warrants discussion about the generalization of our findings. Regarding issues of external validity, a common approach would have been to use observational field data to test the predictions of the theoretical model. However, those data are not available and even if we had data on customer’s evaluation abilities too many important factors like the market environment, the reputation systems, and the particularities of the services would be beyond the control of the researcher to allow for clean causal relationships. In contrast, our experimental design abstracts away from this contextual plurality and isolates the decision-making process of the service provider. This was also the reason to have no human subject in the role of the customer. We avoided that first- and second-order beliefs about the customer’s rating ability biased the behavior of the service provider in an uncontrolled way.

We understand our current investigations as one step to better understand the strategic behavior of service providers. There are several aspects that surely deserve further attention. One is to regard the situation where service providers can enter and exit the market by a costless change of identity [46]. This question is especially interesting for the efficiency of reputation systems in online markets. In this case service providers have strong incentives to behave opportunistically, milk the good reputation until prices go down to a certain threshold, and then change the identity and re-enter the market with a neutral reputation profile. Varying the length of the rating history, weighing the most recent ratings differently or changing the prices asymmetrically between the reception of positive or negative ratings are further determinants of strategic behavior that should be investigated in detail. In addition, the interaction between different service providers directly competing with each other or along a supply-chain with service suppliers and service providers as intermediaries are of particular interest. This extends our current model from a dynamic optimization problem to a stochastic game, which shall be theoretically and experimentally examined in further studies.

## Ethical statement

The data of the study are generated from economic experiments where university students of different majors take economic decisions. The study does not involve any patients or medical treatments. The student participants are recruited from a large subject pool consisting of subjects who signed up for taking part in economic decision laboratory experiments conducted at Paderborn University. When signing up for an experiment the first time (which might not be identical with the present one), subjects are informed about the ethical standards in the experimental economics profession that bans deception. In addition, subjects learn about the specific rules at Paderborn University, in particular concerning the payment for participating in experiments and the measures taken to respect of anonymity of personal data when analyzing the data generated during the experiments. Afterwards, each subject provides a written agreement to take part in experiments conducted at Paderborn University. The experimental procedures of the study in question does not involve any deception of participants. No biological samples are taken. Participants are not expected to suffer and they are not exposed to physical, psychological, financial or legal risks. The participants have the opportunity to earn up to 18 Euro during one regular experimental session. Participants and their data remain anonymous: neither the experimenters nor other participants will be able to trace behavior back to individual participants. Participants in the experiment can terminate the experiment at any time by interrupting the connection to the server and leaving the experimental session. The experimental procedures are in line with the standards of experimental economic research.

Given these preconditions, we did not seek approval of an ethics committee in this case.

## Supporting information

S1 Appendix Proofs, tables, instructions. (PDF)
