# Supplementary material for: Maintaining vs. milking good reputation when customer feedback is inaccurate
Source: PLoS One. 2018 Nov 12;13(11):e0207172. doi: 10.1371/journal.pone.0207172 (PMC6231659; doi:10.1371/journal.pone.0207172)
Supplement: S1 Appendix — (PDF) [file pone.0207172.s001.pdf]

Supporting Information:

Maintaining vs. milking good reputation

when customer feedback is inaccurate

Behnud Mir Djawadi, René Fahr, Claus-Jochen Haake, Sonja Recker

S1 Appendix

Formal description of the Markovian Decision Process and k-responsive Strategies

We give rigorous definitions of the stationary MDP that describes the service provider's repeated decision problem and strategic behavior therein. Generally, a stationary MDP is described by a tuple (S, A, q, π, δ). S is a finite set of states, A a finite set of actions. Transition probabilities are described by the function $q : S \times S \times A \to [0, 1]$ with $\sum_{s' \in S} q(s', s, a) = 1$: i.e., $q(s', s, a)$ is the probability that the process migrates to state s' when the current state is s and action a is taken. Phrased differently, $q(\cdot, s, a)$ is a probability distribution over states conditioned on current state s and action a. To emphasize conditional probabilities, we write $q(s'|s, a)$ instead of $q(s', s, a)$. The function $\pi : S \times A \to \mathbb{R}$ takes each pair of (current) state and action to a payoff to the decision maker describing immediate rewards. That means, $\pi(s, a)$ is the payoff when choosing action $a \in A$ at current state $s \in S$. Finally, $\delta \in (0, 1)$ denotes the decider's discount factor. In what follows, $t = 0, 1, 2, \ldots$ denotes the time index. Stationarity of the MDP in particular means that all ingredients of the MDP are independent of time and need not be indexed with t.

We now specify the general model for our purposes. A state is a vector of the r most recent customer ratings, i.e., the state space is $S = \{+, -\}^r$. At each time t the service provider decides on either producing a service in high (H) or low (L) quality at a cost of c_H or c_L, respectively. Hence, the set of actions is $A = \{H, L\}$. Transition probabilities q depend on the rating probabilities α and β. For any state $s \in S$ there are exactly two possible next states, depending on whether a positive or negative rating is given by the customer (see Fig 1). Denoting by s^+ (s^-) the state that results from a positive (negative) rating, we have $q(s^+|s, H) = \alpha$, $q(s^+|s, L) = \beta$, $q(s^-|s, H) = 1 - \alpha$, and $q(s^-|s, L) = 1 - \beta$.

To specify immediate rewards π, we first formalize the dependency of the sales price on reputation profiles. Let s_i for $i = 1, 2, 3$ denote the ith entry of the reputation profile s and let $\sigma^+(s) := |\{i : s_i = +\}|$ and $\sigma^-(s) := |\{i : s_i = -\}|$ be the numbers of positive and negative

ratings in state s, respectively. Further, define $\sigma(s) := \sigma^+(s) - \sigma^-(s)$. Note that $\sigma(s)$ can only carry values in $\{3, 1, -1, -3\}$. The sales price $P(s)$ in state s is given by $P(s) = \bar{P} + \gamma\sigma(s)$ with a fixed base price $\bar{P}$ and a positive constant γ (price increment). Then the immediate reward (from sales) of the service provider depends on the current state and chosen action and is given by $\pi(s, H) = P(s) - c_H$ and $\pi(s, L) = P(s) - c_L$.

A pure Markovian strategy (or policy) $F = (f_t)_{t\in\mathbb{N}}$ specifies for each period t and each state s an action $f_t(s)$ that the service provider takes, when s is the current state in period t, i.e., $f_t : S \to A$. A Markovian strategy is stationary if $f_t = f_{t+1}$ for all t, i.e., if the chosen actions only depend on the state but not on the time. For a pure Markovian strategy $F = (f_t)_{t\in\mathbb{N}}$ define $Q(f_t)$ to be the $|S| \times |S|$ matrix containing the transition probabilities and $\pi(f_t)$ to be the vector in $\mathbb{R}^{|S|}$ of immediate rewards induced by the actions in f_t for all periods t. More precisely, the entry in $Q(f_t)$ at (s, s') is given by $q(s'|s, f_t(s))$, while the coordinate corresponding to state s in $\pi(f_t)$ is $\pi(s, f_t(s))$. Denote by $Q_t(F) = Q(f_1)\cdots Q(f_t)$ the product of transition matrices until t. The vector in $\mathbb{R}^{|S|}$ of discounted expected profit from using the pure Markovian strategy $F = (f_t)_{t\in\mathbb{N}}$ is given by $\Pi(F) = \sum_{t=0}^{\infty} \delta^t Q_t(F)\pi(f_{t+1})$. If F is a stationary pure Markovian strategy, i.e., $F = (f)_{t\in\mathbb{N}}$ it simplifies to $\Pi(F) = \sum_{t=0}^{\infty} \delta^t (Q(f))^t \pi(f) = (I - \delta Q(f))^{-1}\pi(f)$.

From [20, Corollary of Theorem 3] we know that there exists an optimal pure Markovian strategy that is stationary, and which can be determined by means of an algorithm. To verify whether a stationary strategy $F^* = (f^*)_{t\in\mathbb{N}}$ is optimal, we only need to ensure that for all $g : S \to A$ the inequality $\Pi(F^*) \geq \Pi((g, F^*)) = \pi(g) + \delta Q(g)\Pi(F^*)$ holds, where (g, F^*) is the strategy in which the decision maker deviates from the actions specified in f^* to g in the first period and uses f^* in all subsequent rounds. The inequality says that such one-time deviations do not pay off. This finding is used in the proof of Proposition 1.

Finally, we give a precise definition of k-responsive strategies. For $\ell \in \{1, 2, 3\}$ and $k \in \{0, ..., \ell + 1\}$, a stationary pure Markovian strategy f is *k-responsive to negative ratings for length ℓ* if

$$f_t(s) = \begin{cases} H, & \text{if } \sigma_\ell^-(s) \geq k \\ L, & \text{if } \sigma_\ell^-(s) < k \end{cases}$$

for all t with $\sigma_\ell^-(s) := |\{i \in \{3 - \ell + 1, ..., 3\} : s_i = -\}|$ denoting the number of negative ratings in s among the ℓ most recent ratings.

Proof of Proposition 1

Let $F = (f)_{t\in\mathbb{N}}$ denote a stationary pure Markovian strategy that is independent of the state: i.e., the values $f(s)$ are all either "H" or "L" for any state s. Hence, the probability to obtain a positive rating is given by p_f and the probability for a negative rating is $1 - p_f$. Precisely, $p_f = \alpha$, if $f(s) = H$ for all s and $p_f = \beta$, if $f(s) = L$ for all s.

Suppose the 8 different states are ordered as follows

$$(+++, ++-, +-+, -++, +--, -+-, --+, ---)\,.$$

Then, the transition matrix is

$$Q(f) = \begin{pmatrix} p_f & 1-p_f & 0 & 0 & 0 & 0 & 0 & 0 \\ 0 & 0 & p_f & 0 & 1-p_f & 0 & 0 & 0 \\ 0 & 0 & 0 & p_f & 0 & 1-p_f & 0 & 0 \\ p_f & 1-p_f & 0 & 0 & 0 & 0 & 0 & 0 \\ 0 & 0 & 0 & 0 & 0 & 0 & p_f & 1-p_f \\ 0 & 0 & p_f & 0 & 1-p_f & 0 & 0 & 0 \\ 0 & 0 & 0 & p_f & 0 & 1-p_f & 0 & 0 \\ 0 & 0 & 0 & 0 & 0 & 0 & p_f & 1-p_f \end{pmatrix}.$$

Lemma 1 (Powers of the Transition Matrix)**.** *The sequence of powers of the transition matrix for a state-independent stationary pure Markovian strategy is constant after three periods.*

Proof of Lemma 1. The entries of the transition matrix are the probabilities to move from one state to another within exactly one time step. Similarly, if we consider n time steps then the entries of the nth power of the transition matrix are the probabilities to move in exactly n time steps from one state to another. As the considered strategy is independent of the state, we have by construction in every state an identical probability to obtain a positive rating. Therefore, in exactly n time steps for $n \geq 3$ we are able to get every reputation profile

containing three specified ratings with the following probabilities:

$$Q^n(f)(s,+++) = p_f^3,\quad Q^n(f)(s,++-) = p_f^2\,(1-p_f)\,,$$
$$Q^n(f)(s,+-+) = p_f^2\,(1-p_f)\,,\quad Q^n(f)(s,-++) = p_f^2\,(1-p_f)\,,$$
$$Q^n(f)(s,+--) = p_f\,(1-p_f)^2\,,\quad Q^n(f)(s,-+-) = p_f\,(1-p_f)^2\,,$$
$$Q^n(f)(s,--+) = p_f\,(1-p_f)^2\,,\quad Q^n(f)(s,++-) = (1-p_f)^3$$

for $s \in S$. □

Proof of Proposition 1. Let $F_H = (f_H)_{t\in\mathbb{N}}$ denote the stationary pure Markovian strategy where the service provider always chooses quality H. Then, the immediate reward and the transition matrix are given by

$$\pi(f_H) = \begin{pmatrix} \bar{P}+3\gamma-c_H \\ \bar{P}+1\gamma-c_H \\ \bar{P}+1\gamma-c_H \\ \bar{P}+1\gamma-c_H \\ \bar{P}-1\gamma-c_H \\ \bar{P}-1\gamma-c_H \\ \bar{P}-1\gamma-c_H \\ \bar{P}-3\gamma-c_H \end{pmatrix},\ Q(f_H) = \begin{pmatrix} \alpha & 1-\alpha & 0 & 0 & 0 & 0 & 0 & 0 \\ 0 & 0 & \alpha & 0 & 1-\alpha & 0 & 0 & 0 \\ 0 & 0 & 0 & \alpha & 0 & 1-\alpha & 0 & 0 \\ \alpha & 1-\alpha & 0 & 0 & 0 & 0 & 0 & 0 \\ 0 & 0 & 0 & 0 & 0 & 0 & \alpha & 1-\alpha \\ 0 & 0 & \alpha & 0 & 1-\alpha & 0 & 0 & 0 \\ 0 & 0 & 0 & \alpha & 0 & 1-\alpha & 0 & 0 \\ 0 & 0 & 0 & 0 & 0 & 0 & \alpha & 1-\alpha \end{pmatrix},$$

$$(Q(f_H))^2 = \begin{pmatrix} \alpha^2 & \alpha(1-\alpha) & \alpha(1-\alpha) & 0 & (1-\alpha)^2 & 0 & 0 & 0 \\ 0 & 0 & 0 & \alpha^2 & 0 & \alpha(1-\alpha) & \alpha(1-\alpha) & (1-\alpha)^2 \\ \alpha^2 & \alpha(1-\alpha) & \alpha(1-\alpha) & 0 & (1-\alpha)^2 & 0 & 0 & 0 \\ \alpha^2 & \alpha(1-\alpha) & \alpha(1-\alpha) & 0 & (1-\alpha)^2 & 0 & 0 & 0 \\ 0 & 0 & 0 & \alpha^2 & 0 & \alpha(1-\alpha) & \alpha(1-\alpha) & (1-\alpha)^2 \\ 0 & 0 & 0 & \alpha^2 & 0 & \alpha(1-\alpha) & \alpha(1-\alpha) & (1-\alpha)^2 \\ \alpha^2 & \alpha(1-\alpha) & \alpha(1-\alpha) & 0 & (1-\alpha)^2 & 0 & 0 & 0 \\ 0 & 0 & 0 & \alpha^2 & 0 & \alpha(1-\alpha) & \alpha(1-\alpha) & (1-\alpha)^2 \end{pmatrix}.$$

Applying Lemma 1 the infinite horizon payoff for strategy f_H is given by

$$\Pi\left(F_H\right) = \left(I + \delta Q(f_H) + \delta^2 \left(Q(f_H)\right)^2 + \frac{\delta^3}{1-\delta}\left(Q(f_H)\right)^3\right)\pi(f_H). \tag{1}$$

For a stationary pure Markovian strategy $G = (g)_{t\in\mathbb{N}}$ let $Q(g)$ denote the transition matrix, where p_{g_k} is the probability for a positive evaluation, when s is the state corresponding to row k and action $g(s) \in \{H, L\}$ is played, and let $\pi(g)$ be the immediate reward, i.e.,

$$Q(g) = \begin{pmatrix} p_{g_1} & 1-p_{g_1} & 0 & 0 & 0 & 0 & 0 & 0 \\ 0 & 0 & p_{g_2} & 0 & 1-p_{g_2} & 0 & 0 & 0 \\ 0 & 0 & 0 & p_{g_3} & 0 & 1-p_{g_3} & 0 & 0 \\ p_{g_4} & 1-p_{g_4} & 0 & 0 & 0 & 0 & 0 & 0 \\ 0 & 0 & 0 & 0 & 0 & 0 & p_{g_5} & 1-p_{g_5} \\ 0 & 0 & p_{g_6} & 0 & 1-p_{g_6} & 0 & 0 & 0 \\ 0 & 0 & 0 & p_{g_7} & 0 & 1-p_{g_7} & 0 & 0 \\ 0 & 0 & 0 & 0 & 0 & 0 & p_{g_8} & 1-p_{g_8} \end{pmatrix}$$

and

$$\pi(g) = \begin{pmatrix} \bar{P} + 3\gamma - c_{g_1} \\ \bar{P} + 1\gamma - c_{g_2} \\ \bar{P} + 1\gamma - c_{g_3} \\ \bar{P} + 1\gamma - c_{g_4} \\ \bar{P} - 1\gamma - c_{g_5} \\ \bar{P} - 1\gamma - c_{g_6} \\ \bar{P} - 1\gamma - c_{g_7} \\ \bar{P} - 3\gamma - c_{g_8} \end{pmatrix}$$

In the following, we show that for all $g : S \longrightarrow A$

$$\Pi\left(F_H\right) \geq \Pi\left((g, F_H)\right) = \pi(g) + \delta Q(g)\Pi\left(F_H\right) \tag{2}$$

holds, if and only if $(\star)$ is satisfied. Here (g, f_H) is the strategy in which g is played in the first period and f_H is played in all subsequent periods.

We obtain from combining (1) and (2) and using $Q(g)\left(Q(f_H)\right)^3 = \left(Q(f_H)\right)^3$ the following

$$\begin{aligned}
&\Pi\left(F_H\right) - \pi(g) - \delta Q(g)\Pi\left(F_H\right) \\
&= -\pi(g) + \left(I - \delta Q(g)\right)\left(I + \delta Q(f_H) + \delta^2\left(Q(f_H)\right)^2 + \frac{\delta^3}{1-\delta}\left(Q(f_H)\right)^3\right)\pi(f_H) \\
&= \pi(f_H) - \pi(g) + \delta\left(Q(f_H) - Q(g)\right)\left(I + \delta Q(f_H) + \delta^2\left(Q(f_H)\right)^2\right)\pi(f_H). \qquad (3)
\end{aligned}$$

Note that the entries in each row of the matrix $Q(f_H)$ sum up to one and in each row of $Q(f_H) - Q(g)$ to zero. By writing

$$\pi(f_H) = \left(\bar{P} - c_H\right)e + \begin{pmatrix} +3\gamma \\ +1\gamma \\ +1\gamma \\ +1\gamma \\ -1\gamma \\ -1\gamma \\ -1\gamma \\ -3\gamma \end{pmatrix} \quad \text{with } e = \begin{pmatrix} 1 \\ 1 \\ 1 \\ 1 \\ 1 \\ 1 \\ 1 \\ 1 \end{pmatrix}$$

we obtain

$$\begin{aligned}
&\delta\left(Q(f_H) - Q(g)\right)\left(I + \delta Q(f_H) + \delta^2\left(Q(f_H)\right)^2\right)\left(\bar{P} - c_H\right)e \\
&= \delta\left(\bar{P} - c_H\right)\left(1 + \delta + \delta^2\right)\left(Q(f_H) - Q(g)\right)e = 0 \qquad (4)
\end{aligned}$$

Therefore, we look at

$$\left(I+\delta Q(f_H)+\delta^2 Q^2(f_H)\right)\begin{pmatrix}+3\gamma\\+1\gamma\\+1\gamma\\+1\gamma\\-1\gamma\\-1\gamma\\-1\gamma\\-3\gamma\end{pmatrix}=\gamma\begin{pmatrix}3+\delta\left(1+2\alpha\right)-\delta^2\left(1-4\alpha\right)\\1-\delta\left(1-2\alpha\right)-\delta^2\left(3-4\alpha\right)\\1-\delta\left(1-2\alpha\right)-\delta^2\left(1-4\alpha\right)\\1+\delta\left(1+2\alpha\right)-\delta^2\left(1-4\alpha\right)\\-1-\delta\left(3-2\alpha\right)-\delta^2\left(3-4\alpha\right)\\-1-\delta\left(1-2\alpha\right)-\delta^2\left(3-4\alpha\right)\\-1-\delta\left(1-2\alpha\right)-\delta^2\left(1-4\alpha\right)\\-3-\delta\left(3-2\alpha\right)-\delta^2\left(3-4\alpha\right)\end{pmatrix}. \tag{5}$$

Thus, using (4) and (5) to simplify (3) we have for $g=f_L$

$$\Pi\left(F_H\right)-\pi(f_L)-\delta Q(f_L)\Pi\left(F_H\right)=\begin{pmatrix}c_L-c_H\\c_L-c_H\\c_L-c_H\\c_L-c_H\\c_L-c_H\\c_L-c_H\\c_L-c_H\\c_L-c_H\end{pmatrix}+2\gamma\delta\begin{pmatrix}(\alpha-\beta)\left(1+\delta+\delta^2\right)\\(\alpha-\beta)\left(1+\delta+\delta^2\right)\\(\alpha-\beta)\left(1+\delta+\delta^2\right)\\(\alpha-\beta)\left(1+\delta+\delta^2\right)\\(\alpha-\beta)\left(1+\delta+\delta^2\right)\\(\alpha-\beta)\left(1+\delta+\delta^2\right)\\(\alpha-\beta)\left(1+\delta+\delta^2\right)\\(\alpha-\beta)\left(1+\delta+\delta^2\right)\end{pmatrix},$$

which means that independent of the current state, switching to action L does not strictly pay off, if and only if $c_L-c_H+2\gamma\delta\left(\alpha-\beta\right)\left(1+\delta+\delta^2\right)\geq 0$ holds, which is equivalent to ($\star$). □

Responsive stationary pure Markovian strategies

The k-responsive strategies for reputation profiles consisting of the last three ratings are illustrated graphically in Fig A and analytically in Table A.

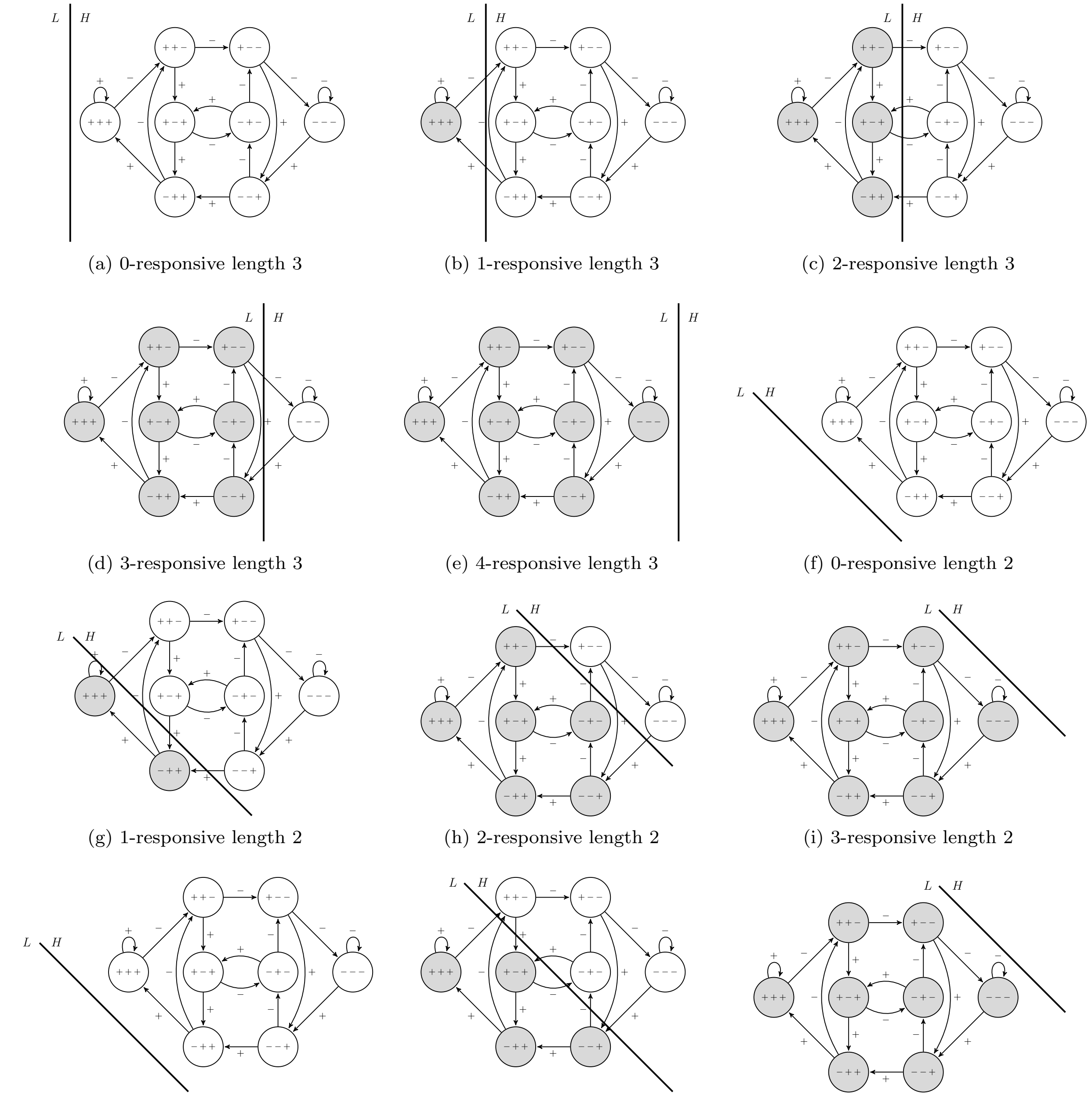

(a) 0-responsive length 3
(b) 1-responsive length 3
(c) 2-responsive length 3
(d) 3-responsive length 3
(e) 4-responsive length 3
(f) 0-responsive length 2
(g) 1-responsive length 2
(h) 2-responsive length 2
(i) 3-responsive length 2
(j) 0-responsive length 1
(k) 1-responsive length 1
(l) 2-responsive length 1

Fig A. Strategies, that are k-responsive (to negative ratings)

Table A. Strategies, that are k-responsive (to negative ratings)

reputation profile	(+++)	(++−)	(+−+)	(−++)	(+−−)	(−+−)	(−−+)	(−−−)
0-responsive, length: 3	H	H	H	H	H	H	H	H
1-responsive, length: 3	L	H	H	H	H	H	H	H
2-responsive, length: 3	L	L	L	L	H	H	H	H
3-responsive, length: 3	L	L	L	L	L	L	L	H
4-responsive, length: 3	L	L	L	L	L	L	L	L
0-responsive, length: 2	H	H	H	H	H	H	H	H
1-responsive, length: 2	L	H	H	L	H	H	H	H
2-responsive, length: 2	L	L	L	L	H	L	L	H
3-responsive, length: 2	L	L	L	L	L	L	L	L
0-responsive, length: 1	H	H	H	H	H	H	H	H
1-responsive, length: 1	L	H	L	L	H	H	L	H
2-responsive, length: 1	L	L	L	L	L	L	L	L

Results of logistic regression for the presence of milking strategies (selected subject sample)

Besides the regressions for the analysis of the complete sample we also run them for the sub-samples which – according to the Brier score – play the reported strategy. When focusing on this sub-sample of subjects who pursued the milking strategies the estimates are even more pronounced.

Table B. Results of logistic regression for the presence of milking strategies (selected subject sample)

dependent variable: high quality	**T1**,**T2**,**T3**					**T4**				
1-resp 1	0.102***<br>(0.048)					–				
1-resp 2		0.010***<br>(0.008)					0.049***<br>(0.019)			
1-resp 3			0.008***<br>(0.006)					0.003***<br>(0.003)		
2-resp 2				0.005**<br>(0.013)					–	
2-resp 3					0.137***<br>(0.095)					–
**T2**	–	1.05<br>(0.274)	1.331<br>-1.305	–	–					
**T3**	0.949<br>(0.140)	0.104***<br>(0.064)	1.443<br>(0.618)	5.63***<br>(3.41)	–					
period	0.982<br>(0.014)	0.987<br>(0.011)	0.983<br>(0.023)	0.903***<br>(0.002)	0.964<br>(0.058)	–	0.995<br>(0.010)	0.976<br>(0.015)	–	–
subjects	5	9	17	2	2	1	6	4	0	1
observations	200	360	680	80	80	40	240	160	0	40

Note: Odds ratios were calculated, robust standard errors are reported in parentheses. Missing results due to low sample size are denoted by "–". Significance at the 1%, 5%, and 10% level is denoted by ***, ** and *, respectively.

Instructions (English version)

The instructions for all treatments and the questionnaire were originally in German. The English instructions presented here are a translation of the originally used ones. In each session the experiment was conducted twice with two different treatments. The sessions have been organized in four different constellations: That is **T1/T3**, **T3/T1**, **T2/T4** and **T4/T2**. This procedure implies that we have observations for each treatment in the first and second position. **For our entire data analysis we only use the observations of the treatments in the first position.** The terms stated in brackets [ ] are values that were modified for different treatments.

General instructions

Today, two experiments will be carried out. The experiments are independent of each other. Hence, your decisions in experiment 1 do not influence your payments in experiment 2 and vice versa. Only one of the experiments will be paid. First, you will receive just the instructions for experiment 1. After its completion we will distribute the instructions for experiment 2. After both experiments are finished, we would like to ask you to fill out a questionnaire. For this you will receive a short instruction as soon as both experiments will have ended. For your responses there is no “right” or “wrong”. The answers of the questionnaire have no influence on your payment.

Payments:

After both experiments are finished there will be a random decision on which of the experiments will be paid. One participant of the room will be asked to throw a dice representatively for all participants. In case of a 1, 2, and 3 all decisions from experiment 1 will be paid for all participants; whereas in case of a 4, 5, and 6 all decisions from experiment 2 will be paid for all participants. The according payments are explained in the related instructions.

Please note:

- During the entire experiment, any form of communication is not permitted.
- All mobile phones must be switched off during the complete duration of the experiment.
- The decisions you make within this experiment are anonymous: i.e., none of the other participants gets to know the identity of a person who has made a specific decision.
- Please remain seated until the end of the experiment.

- You will be called forward for your payment by your seat number.

Good luck and thank you for your participation in this experiment!

Instructions for experiment [1/2]

- During the experiment all payments are stated in the fictitious currency “Taler”.
- Your initial credit is 225 Taler.
- The experiment consists of at least 40 and at most 45 periods. At the beginning of period 41 until period 45 the experiment either continues with a probability of 50% or is terminated with a probability of 50%. This is decided by fair dice. In case of a 1, 2 or 3 the experiment continues and in case of a 4, 5 or 6 the experiment ends. The number rolled on the dice will be displayed on the screen.
- In none of the periods does your payment depend on the decisions of the other participants.

Course of the experiment

Imagine you are a service provider and in each period you sell a service that you produce yourself. In each period you decide if you produce your service in high or low quality. Producing high quality costs you 50 Taler. Producing low quality costs you 35 Taler. In each period there is a customer who wants to purchase a service with high quality. Your payment in one period is computed as follows: The price that you receive from your customer, less the costs of your service that you produced in either high or low quality. Since your customer cannot evaluate the quality of your service in advance, your price depends on the ratings of your previous customers. Your customer in the actual period considers the ratings that you received in the previous three periods. Depending on the number of positive rations (between 0 and 3) your customer is willing to pay a certain price. The more positive ratings you have the higher this price is.

Prices	
Number of positive ratings (within the last 3 periods)	Price that your customer is willing to pay.
3	75 Taler
2	65 Taler
1	55 Taler
0	45 Taler

The initial ratings in period 1 are

(positive, negative, positive).

Therefore, your first customer in period 1 is willing to pay a price of 65 Taler. After your decision to produce your service in high or low quality, your customer receives your service and rates it. Your customer rates your service as either positive or negative. The customer's rating is affected by errors. If you sell a service of high quality to your customer, you receive with a probability of [95%/95%/70%/70%] a positive rating. With a probability of [5%/5%/30%/30%] the rating is negative even if the service's quality was high. If you sell a service of low quality to your customer, you receive with a probability of [95%/70%/95%/70%] a negative rating. With a probability of [5%/30%/5%/30%] the rating is positive even if the service's quality was low. The price that you receive in the next period from your customer depends again on the ratings of the previous three periods.

Example:

Suppose you are in period 4 and you received the following ratings in period 1 to 3:

(period 1, period 2, period 3)=(positive, negative, positive).

In this case the current customer from period 4 is willing to pay a price of 65 Taler for your service. If you receive a positive rating afterwards, the price for the next period is as well 65 Taler. The reason is that the ratings of the last three periods are now

(period 2, period 3, period 4)=(negative, positive, positive).

If you receive a negative rating from your customer, the price for the next period is now 55 Taler on the basis of the last three periods

(period 2, period 3, period 4)=(negative, positive, negative).

In each period the ratings of the last three periods are displayed.

Payments for experiment [1/2]

If experiment [1/2] is paid, the sum of payments over all periods will be converted with an exchange rate of 0.10 Euro per 10 Taler and will be paid in cash together with a show-up fee of 2.50 Euro.
